# Clinical Characteristics of Children with COVID-19

**DOI:** 10.3934/publichealth.2020022

**Published:** 2020-05-06

**Authors:** Carmen Lok Tung Ho, Peter Oligbu, Olakunle Ojubolamo, Muhammad Pervaiz, Godwin Oligbu

**Affiliations:** 1 Imperial College School of Medicine, Imperial College London, South Kensington, London SW7 2AZ, United Kingdom; 2 University of Benin Teaching Hospital (UBTH), Benin City, Nigeria; 3 Department of Medicine, Queens Hospital, Romford, London; 4 Department of Paediatrics, Dumfries and Galloway, NHS Dumfries and Galloway, Scotland, United Kingdom; 5 Department of Paediatrics, Dr Gray's Hospital, NHS Grampian, Elgin, Scotland, United Kingdom; 6 Paediatric Infectious Diseases Research Group, Institute for Infection and Immunity, St. George's, University of London, United Kingdom

**Keywords:** COVID-19, coronavirus, infectious disease, pandemic, 2019-nCoV, severe acute respiratory syndrome coronavirus 2, paediatric, children, SARS-CoV-2, virus

## Abstract

**Background:**

In December 2019, the infection caused by 2019 novel coronavirus (COVID-19) led to an outbreak in Wuhan, situated in the Hubei Province of China. Following this, there has been a rapid increase in the number of cases. On 12th March 2020, there were over 100,000 confirmed cases and almost 4,300 deaths worldwide. The clinical profile of children with COVID-19 is unknown due to the few number of cases reported. Currently, available data suggest they may have a milder form of illness.

**Methods:**

A review of the literature published from June 2019 to March 2020 was undertaken to evaluate the clinical presentation, management and outcomes of COVID-19 in in children. Data sources included EMBASE, MEDLINE, Cochrane library, ISI Web of Knowledge and references within identified articles.

**Results:**

We identified 303 potential studies, and 295 were excluded for reasons including duplicates, experimental studies and case reports. Eight studies were eligible for inclusion, including a total of 820 paediatric cases of COVID-19. Asymptomatic cases represented 14.3% (n = 117) of the total number of cases identified, and thus the remaining 85.7% (n = 703) experienced symptoms. Fever was the commonest symptom in 53.9% (n = 48) of cases, followed by cough in 39.3% (n = 35) of cases, and rhinorrhoea or pharyngeal congestion in 13.5% (n = 12) of cases. Diarrhoea and sore throats were less common symptoms, 7.9% (n = 7) and 9.0% (n = 8) respectively. Other symptoms, including fatigue, headache and dizziness were rare.

**Conclusion:**

Children are disproportionately affected by COVID-19 and are more likely to run a milder cause of illness following this infection compared to adults. This outbreak only started 3 months ago, therefore, further population wide studies are needed to validate these findings.

## 1. Introduction

In December 2019, the infection caused by 2019 novel coronavirus (COVID-19) was first described as pneumonia of unknown cause in Wuhan, situated in the Hubei Province of China [1]. Since then, there has been a rapid increase in the number of cases over the past months. On 12th March 2020, there were over 100,000 confirmed cases and almost 4,300 deaths worldwide. As a result, the World Health Organisation (WHO) announced that the outbreak of COVID-19 should be regarded as a pandemic [2]. This is not surprising given that the world is now a global village and there is limited preparation for such a pandemic [3]. This virus is air borne, and thus transmitted via respiratory droplets and direct contact. Common symptoms reported so far include dry cough, fever, and myalgia [1].

Many countries worldwide have taken precautionary measures such as raising awareness to frequently sanitise hands, advising those with minor symptoms to self-isolate, and to introduce travel restrictions and social distancing [4]. Despite these measures, the spread of the virus remains uncontrolled. Recent studies indicate that the mean incubation period of COVID-19 is 3 to 5 days, but this could range from 0 to 24 days [5],[6]. The case fatality rate following COVID-19 has been estimated to be as high as 7.2% [7]. This may not have considered the asymptomatic individuals and virtually all were reported in adults and the elderly. Information on the prevalence of COVID-19 in children is very scanty due to very few cases reported in children [8]. It is uncertain as to why there are few paediatric cases considering that children have developing immune systems, and thus should be more vulnerable to the virus. However, as the virus continues to spread, the number of cases in children has been rising significantly [9]. In general, the clinical presentation has been less severe in paediatric cases when compared to adult cases, and the reason for this difference is unknown [8]. In addition, pregnant mothers were also advised to stay indoors, as the long-term and short-term consequences of the virus on the foetus and whether there can be mother-to-child vertical transmission is unknown.

Due to the dearth of evidence and information on COVID-19, WHO has encouraged more research, particularly those involving children and pregnant women to provide a better understanding and overview of the clinical characteristics and natural history of the illness [8]. Therefore, this review of all published literature involving children summarises findings from other studies to increase understanding of the clinical presentation, management and outcomes of COVID-19 in this particular group of patients. It is hoped that the findings of this review will provide clinicians with a robust evidence base to investigate and manage children suspected with COVID-19.

## 2. Methods

### 2.1. Information sources and search strategy

A search strategy was designed to identify studies reporting COVID-19 in children. In this review, any patient under the age of 18 will be considered as a child. We searched MEDLINE, EMBASE, and the Cochrane library from 01 June 2019 to 18 March 2020. The medical subject headings (MeSH) terms used included “coronavirus”, “COVID-19”, “2019-nCoV”, “severe acute respiratory syndrome coronavirus 2”, “child”, “paediatric”, “pediatric”, “infant”, “baby”, “newborn”, “children” and “SARS-CoV-2”. These MeSH terms were used in different combinations. The primary search strategy was (“COVID-19” [All Fields] OR “severe acute respiratory syndrome coronavirus 2” [All Fields] OR “severe acute respiratory syndrome coronavirus 2” [All Fields] OR “2019-nCoV” [All Fields] OR “SARS-CoV-2” [All Fields] OR “2019nCoV” [All Fields] OR ((“Wuhan” [All Fields] AND (“coronavirus” [MeSH Terms] OR “coronavirus” [All Fields]))AND (“infant, newborn” [MeSH Terms] OR (“infant” [All Fields] AND “newborn” [All Fields]) OR “newborn infant” [All Fields] OR “baby” [All Fields] OR “infant” [MeSH Terms] OR “infant” [All Fields]). In addition, reference lists of selected papers were screened to retrieve relevant studies.

### 2.2. Selection of studies

Inclusion criteria required the study to report COVID-19 in a child under the age of 18 years old. This was because, in the UK health care system, the cut-off age for admission to paediatric wards is below 18 years in most hospitals. COVID-19 was diagnosed by positive results from identification of 2019-nCoV nucleic acid using real-time reverse-transcriptase polymerase chain reaction (RT-PCR) assay from nasal or pharyngeal swab specimens or blood samples. Alternatively, genetic sequencing of virus genes from respiratory tract or blood samples, that are highly homologous with 2019-nCoV, was another valid method for diagnosis.

Studies were excluded if they were laboratory, experimental, or animal studies. Letters to the editor, case reports, and commentaries were also excluded due to reasons including high probability of bias and low level of evidence. Two independent reviewers (C.H. and G.O.) screened the title and abstract of papers identified by the electronic searches, evaluating inclusion and exclusion criteria for all papers. Articles that met the inclusion criteria were retrieved and reviewed independently for eligibility.

### 2.3. Quality assessment and data extraction

**Figure 1.**
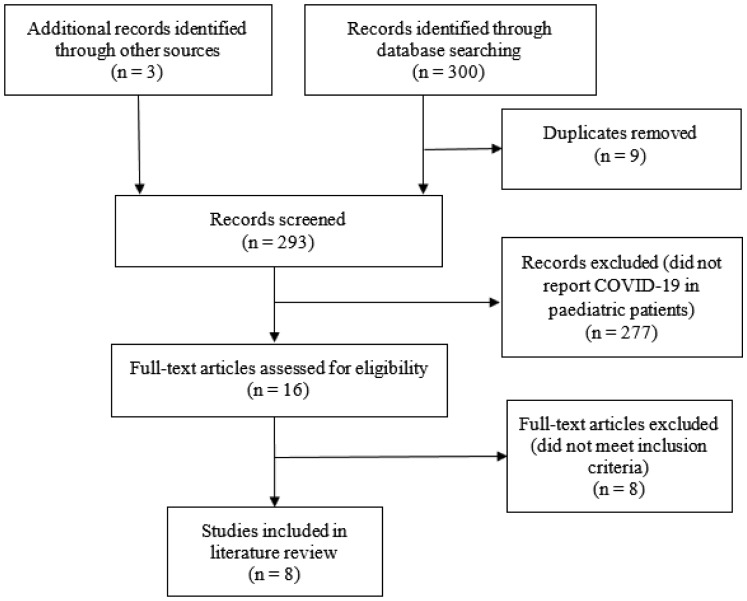
PRISMA flow diagram demonstrating identification and selection of eligible studies for this review.

Two reviewers (C.H. and G.O.) independently reviewed the methodological quality of included studies, comparability of case and controls, and outcomes. Discrepancies were resolved by discussion with a third author (P.O.). The specific variables extracted from the publications included: study design, country, age of participants, year of study, method of data collection, method of diagnosis of COVID-19, whether other causes of respiratory illness, such as the influenza and adenovirus, were excluded, clinical presentation, laboratory results, other abnormal test results, duration of illness, management, and presence of any previous medical issues and outcomes. The study quality assessment for reporting systematic reviews was done according to the Preferred Reporting Items for Systematic Reviews and Meta-analyses (PRISMA) statement [10] (Figure 1).

### 2.4. Data analysis

All studies included in the review were summarised using descriptive analyses to provide an overview of the information on COVID-19 in children in terms of clinical presentations, management, complications and outcome.

## 3. Results

### 3.1. Study characteristics

We identified 303 potential studies during the initial search, of which 10 were duplicates. Of the remaining 293 studies, 277 studies were excluded on the basis of title and abstracts, and a further 8 articles did not meet the inclusion criteria (Figure 1). For example, Guan et al. had paediatric cases, however the data were not separated by age group [6], and consequently data regarding only paediatric cases could not be obtained. Therefore, eight studies were eligible for inclusion in the final analysis [9],[11]–[17]. Of the included studies, all were case series, and these were all published in the year of 2020. All identified cases were patients diagnosed and treated in China. The study by Dong et al. included 2143 patients, however 1412 (65.9%) of the patients were suspected cases and therefore were not confirmed by laboratory results [9]. In addition, the suspected cases had more severe symptoms, and subsequently the authors are of the opinion that these cases may have been caused by alternative respiratory conditions. Thus, only the remaining 731 confirmed cases were included in this study. This study, by Dong et al., did not explicitly describe the clinical characteristics of the confirmed paediatric cases. However, this study provided valuable information on demographics, asymptomatic carriage, diagnostic methods and outcome of cases. A summary of the demographics of the study subjects, method of diagnosis, management and outcome is presented in Tables 1, 2 and 3.

There was a total of 820 paediatric cases of COVID-19 with a mean age of 7 years and 3 months (range of 1 day to 17 years). This average does not include the ages for the patients from the study by Xia et al. and Dong et al., as they only provided the median age, which was 2.125 and 10 years respectively. Out of the 820 patients, 466 (56.8%) were male and 354 (43.2%) were female. All cases, except the cases from Dong et al., were confirmed using nasopharyngeal and oropharyngeal swab specimens to identify the COVID-19 ribonucleic acid (RNA) by the RT-PCR assay. The cases from Dong et al. were confirmed using genetic sequencing of virus genes from respiratory tract or blood samples that were highly homologous with 2019-nCoV (Table 1).

Asymptomatic cases represented 14.3% (n = 117) of the total number of cases identified, and thus the remaining 85.7% (n = 703) experienced symptoms. Excluding information from the study by Dong et al. increases the asymptomatic carrier rate to 25.8% (n = 23/82). Data on clinical presentation was unavailable from the study by Dong et al. Thus, detailed clinical presentations were only available in 7 studies (n = 89), of which fever was the most reported symptom in 53.9% (n = 48) of cases. Most studies did not specify the peak and the duration of fever. The second most common symptom was cough in 39.3% (n = 35) of cases, although majority of the studies did not specify whether the patients experienced a dry or productive cough. In the study by Wang et al., the proportion of patients with dry cough (57.1%, n = 8/14) was larger than for those with productive cough (42.9%, n = 6/14). Rhinorrhoea or pharyngeal congestion was reported in 13.5% (n = 12) of cases, diarrhoea in 7.9% (n = 7) and sore throats in 9.0% (n = 8) of cases. Other symptoms, such as headache or dizziness (3.4%, n = 3) and fatigue (4.5%, n = 4) were rarely reported (Table 3). Interestingly, Hu et al. described a rash in a 14-year-old female patient. The rash was described as most likely due to a reaction to an infusion of intravenous immunoglobulin or a side effect to the medications prescribed: lopinavir/ritonavir and darunavir/cobicistat. Only Xia et al. reported underlying illnesses in three patients: two had previous history of atrial septal defect surgery and one had epilepsy from previous viral encephalitis.

Laboratory results were reported in 10.1% (n = 83) of cases. White blood cell counts (WBC) was normal in 68.7% (n = 57) of cases, and the remaining 18.1% (n = 15) and 13.3% (n = 11) had low and elevated WBCs respectively. Some studies also reported the levels of C-reactive protein (CRP); this was elevated in 29.9% (n = 20/67) of cases. Most studies included a computed tomography (CT) scan, except for the studies by Dong et al. and Cai et al., which mainly used chest x-rays. In 57.3% (n = 51) of cases the radiological findings were abnormal as there were 4 abnormal chest x-rays and 47 abnormal CT scans. Two studies did not describe the lesion or report the location of the lesion. In the remaining six studies, the lesions were described, and they generally reported to have occurred in the lower zones of the lungs and appeared to be ground-glass opacities (Table 2).

All patients, including those who were asymptomatic, were admitted into hospital and mainly required symptomatic and supportive treatment. Antiviral therapy (n = 52), antibiotics (n = 12), and interferon atomization (n = 6) were also other treatments used. Alternative medicines (n = 10), such as those from traditional Chinese herbal medicine, was also used by Wang et al. and Li et al., such as Lianhuaqingwen granules and oral Yiqi Yangyin decoctions. Wang et al. described one patient with no treatment, but still recovered fully from their fever and any previous respiratory and gastrointestinal symptoms. Feng et al. did not specify the outcome of their patients, and Dong et al. described a 14-year-old who died but did not specify whether they were a suspected or confirmed case of COVID-19. All other cases from the remaining 6 studies survived with no reported mortality.

**Table 1.** Description of study designs and reported COVID-19 in children in the published studies that were included in the review.

Study reference	Year of publication	Country	Study design	Total cases	Paediatric cases	Female paediatric cases	Male paediatric cases	Age range of paediatric patients	Data collection method		Diagnostic method for COVID-19		Co-infections with other respiratory infections
Cai et al.	2020	China (Shanghai)	Case series	10	10	6	4	3 months to 11 years	Between 19th January and 3rd February 2020 in Children's Hospital in Shanghai, Hainan, Hefei in Anhui province, and Qingdao in Shandong province.		Nasopharyngeal/throat swabs, stool specimens, urine and serum samples, and chest x-ray		No
Wang et al.	2020	China (Shaanxi, Gansu, Ningxia, Hebei, Henan and Shandong)	Case series	31	31	16	15	6 months to 17 years	Between 25th January 2020 and 21st February 2020 in 21 hospitals in 17 cities of 6 provinces of Shaanxi, Gansu, Ningxia, Hebei, Henan and Shandong.		Chest CT and qRT-PCR		No
Li-Na et al.	2020	China (Beijing)	Case series	2	2	0	2	9 and 15 years	On 25th January and 3rd February 2020, they were admitted to Beijing Tsinghua Changgung Hospital.		Oropharyngeal swabs for RT-PCR and chest CT		No
Hu et al.	2020	China (Nanjing)	Case series	24	6	3	3	5 to 15 years	Between 28th January to 9th February 2020 in Nanjing, Jiangsu Province, China.		Chest CT and pharyngeal swab specimens for qRT-PCR		No
Xia et al.	2020	China (Wuhan)	Case series	20	20	7	13	1 day to 14.5 years	Between 23rd January and 8th February 2020 in Wuhan Children's Hospital.		Pharyngeal swab samples for RT-PCR and chest CT.		8 had co-infections, including influenza viruses A and B, mycoplasma, respiratory syncytial virus, and CMV.
Feng et al.	2020	China (Shenzhen)	Case series	15	15	10	5	4 to 14 years	Between 16th January to 6th February 2020 in Hospital of Shenzhen		Nasal or pharyngeal swabs for RT-PCR and chest CT.		No
Dong et al.	2020	China	Case series	731	731	311	420	0 to 18 years	Nationwide cases reported to the Chinese Center for Disease Control and Prevention from 16th January to 8th February 2020.		Nasal and pharyngeal swab for RT-PCR or genetic sequencing. Chest CT or chest x-rays were also used.		No
Li et al.	2020	China (Guangdong)	Case series	5	5	1	4	10 months to 6 years	Between 28 January 2019 to 8 February 2020 at the Fifth Affiliated Hospital.		Chest CT and RT-PCR		No

*Notes: Abbreviations: CT, computed tomography; RT-PCR, reverse transcriptase polymerase chain reaction; qRT-PCR, quantitative reverse transcriptase polymerase chain reaction; CMV, cytomegalovirus.

**Table 2.** Clinical characteristics, management and outcome of children with COVID-19 in the published studies that were included in the review.

Study reference	WCC Results	Chest x-ray or CT scan results	Other findings	Duration of diagnosis to recovery	Required hospitalisation	Management methods		Previous medical history	Outcome	No. with contact history	No. with travel history
Cai et al.	Low: 1 Normal: 6 High: 3	Normal: 6 Abnormal: 4	Elevated CRP =3.	N/A	All	All had symptomatic treatment with 5 cases given antibiotics.		N/A	All lived	8	0
Wang et al.	Low: 2 Normal: 26 High: 3	Normal: 17 Abnormal: 14	Elevated CRP = 3 Elevated ESR = 4 Elevated procalcitonin = 4 Liver enzyme = 6 Muscle enzyme = 4	7 to 23 days	All	Mainly supportive treatment. 29 children received antiviral therapy. 6 children with antibacterial drugs, 9 children received symptomatic oral decoction, 1 patient was not treated.		All vaccinated as planned.	All lived	22	9
Li-Na et al.	Low: 0 Normal: 1 High: 1	Normal: 2 Abnormal: 0	Elevated CRP = 1	2 days for both cases	All	1 case was given symptomatic treatment and 1 case with probiotics.		None.	All lived	1	2
Hu et al.	N/A	Normal: 5 Abnormal: 1	Patients with normal CT and no symptoms were younger.	1 to 14 days to recover, while 2 patients still not recovered.	All	All cases were treated with interferon atomization. Antiviral therapy for some cases.		N/A	All lived	0	3
Xia et al.	Low: 4 Normal: 14 High: 2	Normal: 0 Abnormal: 20	Elevated ALT = 5 Elevated CK = 15 Elevated procalcitonin = 16 Abnormal electrocardiogram = 4	18 patients had an average stay of 12.9 days (8–20 days).	All	N/A		2 previously had atrial septal defect surgery, 1 with epilepsy from previous viral encephalitis.	All lived	13	0
Feng et al.	Low: 8 Normal: 7 High: 0	Normal: 6 Abnormal: 9		After 3–5 days of treatment, 6 cases recovered.	All	N/A		N/A	N/A	12	3
Dong et al.	N/A	N/A		N/A	All	N/A		N/A	1 death	N/A	N/A
Li et al.	Low: 0 Normal: 3 High: 2	Normal: 2 Abnormal: 3 (Patchy GGOs)	Elevated CRP = 1	12–14 days for 3 patients and other 2 patients still in hospital.	All	Varied between all cases such as antivirals, anti-infective therapy, immunoglobulin therapy, interferon, and Lianhua qingwen granules.			All lived	4	1

*Notes: Abbreviations: ALT, alanine transaminase; CK, creatinine kinase; CT, computed tomography; CRP, C-reactive protein; ESR, erythrocyte sedimentation rate; GGO, ground glass opacities; WCC, white cell count; N/A, not available.

**Table 3.** Signs and symptoms of children with COVID-19 in the published literature.

Number of cases with type of symptom												
Study reference	Asymptomatic	Fever	Cough	Diarrhoea	Fatigue	Rhinorrhoea	Nasal Congestion	Dyspnoea	Sore Throat	Vomiting	Headache or dizziness	Rashes
Cai et al.	0	8	6	0	0	2	3	0	4	0	0	0
Wang et al.	4	20	14	3	3	2	0	0	2	2	3	0
Li-Na et al.	0	1	0	1	0	0	0	0	0	0	0	0
Hu et al.	5	1	0	0	0	0	0	0	0	0	0	1
Xia et al.	2	12	13	3	1	3	0	0	1	2	0	0
Feng et al.	8	5	1	0	0	0	1	0	0	0	0	0
Dong et al.	94	N/A	N/A	N/A	N/A	N/A	N/A	N/A	N/A	N/A	N/A	N/A
Li et al.	4	1	1	0	0	1	0	0	1	0	0	0

*Notes: Abbreviations: N/A, not available.

Data regarding contact history was provided in all studies, except the study by Dong et al., and 65.2% (n = 58/89) reported to have had physical contact with another confirmed patient. Recent travel was also reported in 11.2% (n = 10) of cases (excluding cases from Dong et al.) where they had been to Wuhan, the epicentre of the outbreak, or other epidemic areas such as Hubei.

## 4. Discussion

A detailed review of all published articles on COVID-19 identified a very low rate in children, accounting for ∼2% (80,900 cases reported in China as of 12th March 2020) [18]. Similar findings were observed in Europe with 1% in children less than 10 years old and 4% in 10 to 19 years had laboratory-confirmed cases for COVID-19 [19]. One in seven confirmed cases in children were asymptomatic, with fever and cough being the commonest presentation in symptomatic cases. Radiological investigations were abnormal in more than half of cases. All cases in children recovered with no reported fatality.

Despite COVID-19 being the cause of the pandemic in 2020, and thus affecting a large population worldwide, very few studies have been published in children to understand the pathophysiology, management and outcome in this cohort. This is not surprising since the first case was only reported in December 2019, and researchers and clinicians are currently heavily occupied with clinical tasks to treat this cohort of patients. Currently, most studies on COVID-19 are in adults and the elderly [8]. Possible explanations for this could include reduced exposure of children to those infected in the community, travellers and clinical areas. Other reasons may be that children have a relatively immature immunity to viruses, and consequently may respond to COVID-19 differently to adults. Recent experimental studies have concluded that, like the severe acute respiratory syndrome coronavirus (SARS-CoV), the novel coronavirus 2019 (2019-nCoV) uses the same receptor: angiotensin converting enzyme II (ACE-II) [20],[21]. It is therefore possible that the activity or function of ACE-II in children is not up to the same standard as in adults. It can also be argued that it is too early to accurately make any reasonable conclusions, and, in the future, there may be a sudden increase in paediatric cases.

It is important to emphasise that there is a high number of asymptomatic cases, as this review have found asymptomatic cases in one in every seven confirmed case. Furthermore, there is a lot of stigma on any individual who report to have minor symptoms regardless of the cause of their illness. Consequently, the proportion of asymptomatic cases of COVID-19 may be higher, as some are unaware or are afraid to contact healthcare services. In addition, there may be fewer children being tested for COVID-19, as from what the current literature suggests, there are more silent and asymptomatic cases in this group of patients [9]. Therefore, more studies are needed to investigate the epidemiology of COVID-19 and the causes of any age-related differences if they exist.

This review found that almost two-third of cases had a physical contact with a confirmed case of COVID-19 suggesting that the mode of transmission for this virus is person-to-person. For example, Chan et al. reported a Chinese family who had travelled from Wuhan and subsequently, five out of six members were infected. Another family member who did not travel to Wuhan, but had been in contact with this family, was infected with COVID-19 [22]. However, the mode of spread and incubation period was not reported by many studies. Cai et al. obtained data concerning the period between symptom onset and exposure to index case, and the median incubation period was 7 days across 8 paediatric patients [13]. A population-level observational study analysed 33 adult patients from Wuhan and the estimated median incubation period was 4.5 days [23]. Similarly, Lauer et al. collected data from 181 confirmed cases, and the median incubation period was 5.1 days. This also demonstrated that 97.5% of patients who do experience symptoms will show symptoms by 11.5 days from the time of exposure [24]. Although relatively similar, the difference in the incubation periods between the paediatric and adult cases could be related to the severity of disease. For example, in another similar outbreak, which occurred in 2003, caused by severe acute respiratory syndrome (SARS), the association between incubation period and severity of disease was analysed. It was observed that the severe cases tended to have a significantly shorter incubation period [25]. Transmission patterns of the disease caused by COVID-19 were comparable with the pattern seen with SARS-CoV [26]. Consequently, given that this review mainly focused on COVID-19 cases in children, who are currently believed to experience less severe symptoms than adults and the elderly [9], the longer incubation period compared to other studies may be accounted for by the milder form of disease in children. However, further studies should specify the incubation period to validate these finding.

Symptoms associated with COVID-19 have so far been non-specific. The most common symptom was fever, followed by cough, in this review. This is consistent with the data from Huang et al., which only included adult and elderly patients, as this showed that fever and cough was a symptom in 98% (n = 40) and 76% (n = 31) respectively of all patients included in their study [27]. The higher prevalence of fever found in their study, when compared to cases in children, may reflect the increased severity of disease in adult patients. However, irrespective of the incubation period, the current WHO guidance recommends laboratory investigations to ascertain cases due to COVID-19. This involves collecting specimens from the upper respiratory tract, including the nasopharyngeal and oropharyngeal regions for all suspected cases of COVID-19 regardless of age. Laboratory investigations should include using RT-PCR and bacterial cultures [8]. All studies, except for Dong et al. where some patients were diagnosed using genetic sequencing, confirmed diagnosis using investigations that aligned with the WHO guidance. The diagnostic test for COVID-19 is the RT-PCR. In this review, only confirmed cases were included. The report from Yang et al. revealed that the sputum and nasal swabs have the potential to achieve a positive rate of 88.9% and 73.3% respectively during the first 14 days after illness onset [28]. As a result, false negatives are possible, which will not only reduce the reported incidence of cases but also be detrimental in contact tracing and containing the spread of this virus. Other investigations used by studies included in this review are CT scans and chest x-rays [9],[11]–[17]. CT scans provide a higher resolution, and therefore may be preferred over x-rays. In comparison, x-rays would be preferred in critically ill patients where only bedside investigations are possible. Given the high resolution of CT, this has become the image of choice in severe cases. This may be particularly useful in children as CT scans would provide a clearer image of small lesions, which are more common in children due to milder forms of disease present, than x-rays. However, clinicians should weigh the benefit of the radiation involved in CT scans against trying to make a radiological diagnosis in a growing child. The radiological finding of ground-glass opacities in the lower zones of the lungs was the most frequent findings in this review. The ‘halo sign’, which is represented by a ground-glass opacity surrounded by a pulmonary nodule or mass, is a relatively rare in adults [29], however Xia et al. found this in 50% (n = 20) of the paediatric patients [16]. In another study with only adult patients, only 3.9% (n = 2) of patients had absence of ground-glass opacities and consolidations, whereas this review estimates this to be 42.6% (n = 38) in children when using the studies that provided this data. This further provides evidence that adults and children present differently, and the explanations for these differences need to be explored further in future studies.

The significance of blood inflammatory markers remains controversial, particularly in children. We observed that over two-third (68.7%, n = 57) of cases had a normal WBC and a normal CRP in 70.1% (n = 47/67) of cases. Whereas in another study by Huang et al., which only involved adult patients, 45% (n = 18) of cases had normal WBCs, with 25% (n = 10) and 30% (n = 12) having low and elevated WBCs respectively [27]. However, in contrary, elevation of procalcitonin was reported in 80% of cases involving children in another study, suggesting that a possible co-infection with bacteria might be common compared with adults with COVID-19 [16]. These children may therefore benefit from additional antimicrobial cover.

More COVID-19 related deaths have been reported in the adult population than in children [27]. Possible explanations for this could be the impaired immunity in the elderly patients, and the increased prevalence of co-morbidities such as cancer, diabetes and cardiovascular diseases in the adult and elderly. As a result, adult and elderly patients are more prone to end organ damage and systemic failures following COVID-19 [27]. In addition, adult patients are more likely to smoke cigarettes, which has been found to be associated with increased risks of acute respiratory distress syndrome (ARDS) [30]. Consequently, ARDS was a complication in 29% (n = 12) of all adult patients in one study [27]. This risk is not clearly understood, thus future studies should focus on age and sex related factors contributing to the outcomes following COVID-19.

Treatment of symptomatic cases have been rather challenging. Current WHO recommendation for all patients with mild symptoms include antipyretics and self-isolation at the patient's home. In this review, despite some cases being asymptomatic, all were hospitalised. This method was adopted primarily to reduce further transmission in the population and not due to clinical effects of COVID-19. For severe cases, including those that experience shock and ARDS, should be hospitalised, and respiratory and cardiovascular support should be provided [8]. Given that there are no specific treatments for COVID-19 due to the lack of current evidence, various combinations of treatments were trailed; this ranged from probiotics [14], antiviral therapy to interferon atomisation [15]. Whether this made a difference to the outcome of these cases is yet to be determined. A prospective and randomised-controlled trials are warranted to develop a specific and detailed treatment guideline for clinicians.

This is a review of COVID-19 cases involving children, however there are limitations to this study. The first case of COVID-19 was reported in China in December 2019, and subsequently in other countries. As a result, most studies reporting cases of COVID-19 were from China, thus, a regional bias may be unavoidable. Further studies are needed to understand if there are differences in presentations and clinical features between children from different countries. A second limitation is that some studies were written in Chinese, and therefore needed interpretation. As a result, the accuracy of interpretation may be questioned. In addition, the study by Dong et al. is a nationwide time limited epidemiological study of all the paediatric patients in China from 16th January to 8th February 2020. Consequentially, there is a chance that few of the patients are duplicated and reported in another study included in this review. However, as the data is non-identifiable, it is difficult to eliminate the duplicate studies. It is also important to note that, given the novelty of COVID-19, there has been few studies that were eligible to be included in this review. Consequently, all studies were case series, which is known to provide a lower level of evidence when compared to other study designs. Nevertheless, this review further highlights important findings in the cases of COVID-19 reported in children. Given that one in seven of all cases were asymptomatic means that contact tracing and testing all physical contacts will help reduce the spread of this novel virus. Until a vaccine is available, reporting cases, and especially those that are outside of China, will be required to monitor the trend of the pandemic.

## 5. Conclusion

The current available data suggests that children are disproportionately affected by COVID-19 and are more likely to run a milder course following this infection with COVID-19 compared to adults. This study also identified a need for standardised international reporting of COVID-19 cases in children to better understand the trend and possible complications associated with this virus. In addition, given that no treatment has been identified, prospective and randomised-controlled trials would be beneficial to provide robust evidence for development of a treatment strategy to reduce current morbidity in children.

## References

[1] Chen N, Zhou M, Dong X (2020). Epidemiological and clinical characteristics of 99 cases of 2019 novel coronavirus pneumonia in Wuhan, China: a descriptive study. Lancet.

[2] World Health Organisation (WHO) (2020). WHO Director-General's opening remarks at the media briefing on COVID-19. https://www.who.int/dg/speeches/detail/who-director-general-s-opening-remarks-at-the-media-briefing-on-covid-19---11-march-2020.

[3] Oligbu G (2019). Rare and Imported Infections: Are We Prepared?. Pharmacy (Basel, Switzerland).

[4] Public Health England Stay at home: guidance for households with possible coronavirus (COVID-19) infection. https://www.gov.uk/government/publications/covid-19-stay-at-home-guidance/stay-at-home-guidance-for-households-with-possible-coronavirus-covid-19-infection.

[5] Li Q, Guan X, Wu P (2020). Early Transmission Dynamics in Wuhan, China, of Novel Coronavirus-Infected Pneumonia. N Engl J Med.

[6] Guan W, Ni Z, Hu Y (2020). Clinical Characteristics of Coronavirus Disease 2019 in China. N Engl J Med.

[7] Onder G, Rezza G, Brusaferro S (2020). Case-Fatality Rate and Characteristics of Patients Dying in Relation to COVID-19 in Italy. JAMA.

[8] Moreton E (2020). Clinical management of severe acute respiratory infection (SARI) when COVID-19 disease is suspected. World Health Organisation.

[9] Dong Y, Mo X, Hu Y (2020). Epidemiological Characteristics of 2143 Paediatric Patients With 2019 Coronavirus Disease in China. Pediatrics.

[10] Moher D, Liberati A, Tetzlaff J (2009). Preferred reporting items for systematic reviews and meta-analyses: the PRISMA statement. BMJ.

[11] Feng K, Yun YX, Wang XF (2020). Analysis of CT features of 15 Children with 2019 novel coronavirus infection. Chin J Contemp Pediatr.

[12] Wang D, Ju XL, Xie F (2020). Clinical analysis of 31 cases of 2019 novel coronavirus infection in children from six provinces (autonomous region) of northern China. Chin J Contemp Pediatr.

[13] Cai J, Xu J, Lin D (2020). A Case Series of children with 2019 novel coronavirus infection: clinical and epidemiological features. Clin Inf Dis.

[14] Ji LN, Chao S, Wang YJ (2020). Clinical features of pediatric patients with COVID-19: a report of two family cluster cases. World J Pediatr.

[15] Hu Z, Song C, Xu C (2020). Clinical characteristics of 24 asymptomatic infections with COVID-19 screened among close contacts in Nanjing, China. Sci China Life Sci.

[16] Xia W, Shao J, Guo Y (2020). Clinical and CT features in pediatric patients with COVID-19 infection: Different points from adults. Pediatric Pulmonol.

[17] Li W, Cui H, Li K (2020). Chest computed tomography in children with COVID-19 respiratory infection. Pediatric Radiol.

[18] Statista Number of novel coronavirus COVID-19 cumulative confirmed and death cases in China from January 20 to April 24, 2020. https://www.statista.com/statistics/1092918/china-wuhan-coronavirus-2019ncov-confirmed-and-deceased-number/.

[19] European Centre for Disease Prevention and Control (2020). Coronavirus disease 2019 (COVID-19) pandemic: increased transmission in the EU/EEA and the UK-seventh update.

[20] Zhou P, Yang X, Wang X (2020). A pneumonia outbreak associated with a new coronavirus of probable bat origin. Nature.

[21] Wrapp D, Wang N, Corbett KS (2020). Cryo-EM structure of the 2019-nCoV spike in the prefusion conformation. Science.

[22] Chan JF, Yuan S, Kok K (2020). A familial cluster of pneumonia associated with the 2019 novel coronavirus indicating person-to-person transmission: a study of a family cluster. Lancet.

[23] Sun K, Chen J, Viboud C (2020). Early epidemiological analysis of the coronavirus disease 2019 outbreak based on crowdsourced data: a population-level observational study. Lancet Digital Health.

[24] Lauer SA, Grantz KH, Bi Q (2020). The Incubation Period of Coronavirus Disease 2019 (COVID-19) From Publicly Reported Confirmed Cases: Estimation and Application. Ann Intern Med.

[25] Virlogeux V, Fang VJ, Wu JT (2015). Incubation Period Duration and Severity of Clinical Disease Following Severe Acute Respiratory Syndrome Coronavirus Infection. Epidemiology.

[26] Riou J, Althaus CL (2020). Pattern of early human-to-human transmission of Wuhan 2019 novel coronavirus (2019-nCoV), December 2019 to January 2020. Eurosurveillance.

[27] Huang C, Wang Y, Li X (2020). Clinical features of patients infected with 2019 novel coronavirus in Wuhan, China. Lancet.

[28] Yang Y, Yang M, Shen C (2020). Evaluating the accuracy of different respiratory specimens in the laboratory diagnosis and monitoring the viral shedding of 2019-nCoV infections. MedRxiv.

[29] Li Y, Xia L (2020). Coronavirus Disease 2019 (COVID-19): Role of Chest CT in Diagnosis and Management. Am J Roentgenol.

[30] Calfee C, Matthay M, Kangelaris K (2015). Cigarette Smoke Exposure and the Acute Respiratory Distress Syndrome. Crit Care Med.

